# In vitro single vesicle fusion assays based on pore-spanning membranes: merits and drawbacks

**DOI:** 10.1007/s00249-020-01479-0

**Published:** 2020-12-15

**Authors:** Peter Mühlenbrock, Merve Sari, Claudia Steinem

**Affiliations:** 1 Georg-August-Universität Göttingen, Institute of Organic and Biomolecular Chemistry, Tammannstr. 2, 37077 Göttingen, Germany; 2 Max-Planck-Institute for Dynamics and Self Organization, Am Faßberg 17, 37077 Göttingen, Germany

**Keywords:** Content mixing, Fluorescence microscopy, Lipid mixing, Model membranes, SNAREs

## Abstract

Neuronal fusion mediated by soluble *N*-ethylmaleimide-sensitive-factor attachment protein receptors (SNAREs) is a fundamental cellular process by which two initially distinct membranes merge resulting in one interconnected structure to release neurotransmitters into the presynaptic cleft. To get access to the different stages of the fusion process, several in vitro assays have been developed. In this review, we provide a short overview of the current in vitro single vesicle fusion assays. Among those assays, we developed a single vesicle assay based on pore-spanning membranes (PSMs) on micrometre-sized pores in silicon, which might overcome some of the drawbacks associated with the other membrane architectures used for investigating fusion processes. Prepared by spreading of giant unilamellar vesicles with reconstituted t-SNAREs, PSMs provide an alternative tool to supported lipid bilayers to measure single vesicle fusion events by means of fluorescence microscopy. Here, we discuss the diffusive behaviour of the reconstituted membrane components as well as that of the fusing synthetic vesicles with reconstituted synaptobrevin 2 (v-SNARE). We compare our results with those obtained if the synthetic vesicles are replaced by natural chromaffin granules under otherwise identical conditions. The fusion efficiency as well as the different fusion states observable in this assay by means of both lipid mixing and content release are illuminated.

## Introduction

### Neuronal fusion and SNAREs

**Fig. 1:**
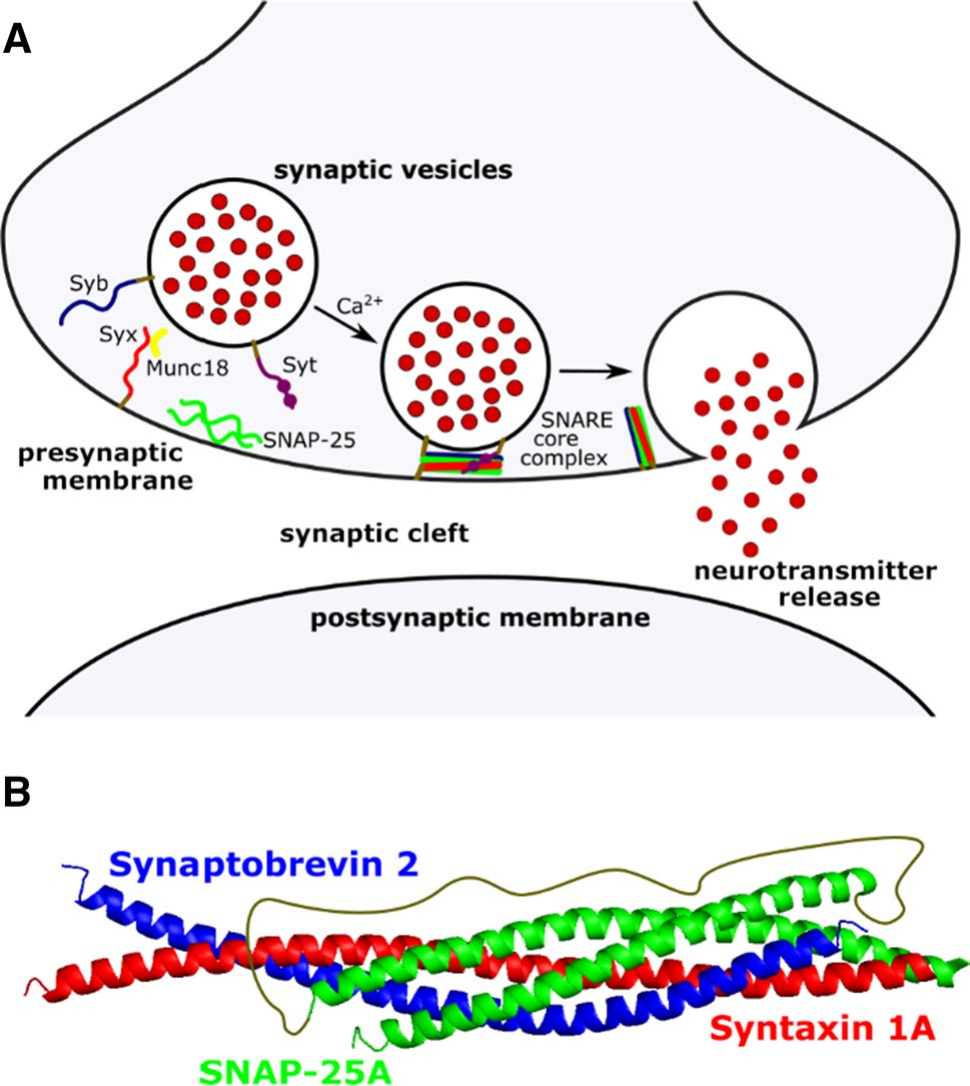
**a** Ca^2+^-triggered synaptic membrane fusion. To transmit the incoming electric signal to the next neuron, neurotransmitters have to be released into the synaptic cleft. Therefore, target-SNAREs (syntaxin 1A (Syx) and SNAP-25) and the vesicle-SNARE (synaptobrevin 2 (Syb)) form the SNARE core complex enabling the fusion of the synaptic vesicle containing the neurotransmitter with the presynaptic membrane. Other proteins that control the fusion processes are also involved such as synaptotagmin 1 (Syt) and Munc18. **b** SNARE core complex. The mainly hydrophobic interaction between the helices of the three SNAREs leads to a coiled coil structured complex with a main site of interaction termed the zero ionic layer (PDB:3HD7)

In eukaryotic cells, synaptic vesicle fusion is one of the pivotal steps during neuronal signal transduction (Brose et al. 2019). An incoming action potential causes the influx of Ca^2+^ ions triggering the fusion of synaptic vesicles with the neuronal presynaptic membrane thus releasing neurotransmitters into the synaptic cleft (Fig. 1a). At the active zone of the synaptic bouton, numerous proteins are found being involved in the exocytosis process (Wilhelm et al. 2014). Among them, the soluble *N*-ethylmaleimide-sensitive-factor attachment protein receptors (SNAREs, Fig. 1) are the main players providing the driving force for docking and fusion of a synaptic vesicle with the presynaptic membrane. The neuronal SNAREs that build up the minimal fusion machinery (Weber et al. 1998) are syntaxin 1A (Bennett et al. 1992) anchored by its transmembrane domain in the presynaptic (target) membrane (t-SNARE) together with SNAP-25 (t-SNARE) (Oyler et al. 1989), which is peripherally attached to the target membrane via palmitoyl side chains covalently bound to cysteine amino acid residues, and synaptobrevin 2 (v-SNARE) (Baumert et al. 1989) localised in the vesicle membrane.

Without complex formation, SNAREs are unstructured monomers (Fasshauer et al. 1997). However, the three SNAREs are capable of forming a stable *trans-*SNARE complex independent of the other proteins that are involved, such as Munc18, synaptotagmin 1 (Syt), α-SNAP and NSF to name just a few (Söllner et al. 1993). This *trans*-SNARE core complex was predicted to be a coiled coil structure (Chapman et al. 1994; Hao et al. 1997; Hayashi et al. 1994) formed via the SNARE motifs with a length of around 60 amino acids (Jahn and Südhof 1999; Weimbs et al. 1997) (Fig. 1b), which was confirmed by the crystal structure (Sutton et al. 1998). The crystal structure further showed a specific hydrophilic interaction between three glutamine residues (Q) (syntaxin 1A and SNAP-25) and one arginine residue (R) (synaptobrevin 2), which was named the zero ionic layer. This observation led to a reclassification of the SNAREs in Q- and R-SNAREs (Fasshauer et al. 1998). It was hypothesised that the SNARE complex is formed via a “zippering” mechanism (Hanson et al. 1997; Harbury 1998; Lin and Scheller 1997), which was later on specified as an *N*- to *C*-terminal “zippering” (Li et al. 2014; Pobbati et al. 2006). “Zippering” of the SNAREs forces the vesicular and target membrane into close proximity, which leads to fusion. The complex formation provides the required energy release needed to overcome the energy barrier that is given by repulsive interactions occurring if the membranes come in close proximity (Fasshauer 2003). After fusion, the SNARE core complex remains in the *cis*-configuration until the proteins are recycled into their original states (Jahn and Fasshauer 2012).

### In vitro fusion assays

Even though quite a number of studies have been performed to elucidate the neuronal fusion process, leading to a number of proposed fusion intermediates (Marsden et al. 2011), there is still a huge demand in developing suitable fusion assays to draw a comprehensive and consistent picture of the fusion process. Besides in vivo assays, in the last decades, numerous in vitro assays were established to characterise the SNAREs, elucidate the SNARE core complex and the fusion process as well as their intermediates.

The first reported in vitro bulk assay to detect SNARE-mediated membrane fusion was developed by Weber et al. (1998). Based on Förster resonance energy transfer (FRET) as reported by Struck et al. (1981), lipid mixing between two vesicle populations, defined as membrane fusion, was measured. In this bulk assay, the t-SNAREs syntaxin 1A/SNAP-25 and the v-SNARE synaptobrevin 2 were reconstituted into small and/or large unilamellar vesicles. Two different approaches were pursued to measure lipid mixing: (i) The membrane of one vesicle population is labelled with a FRET pair, whereas the other one is unlabelled. If lipid mixing occurs, the distance between the fluorophores of the FRET pair increases due to dilution and thus, the donor fluorescence increases (dequenching). (ii) Each membrane of the two vesicle populations is doped with one of the fluorophores forming the FRET pair. If lipid mixing takes place, the acceptor fluorescence increases owing to the now occurring FRET. Besides lipid mixing, also content mixing assays (van den Bogaart et al. 2010) can be performed in bulk, providing additional information about whether hemifusion or full fusion has occurred. However, even though these assays deliver data about fusion efficiencies and average fusion kinetics, several information cannot be read out such as complex fusion intermediates or kinetic aspects such as docking lifetimes. Moreover, in some assays, vesicle aggregation and bursting cannot be readily distinguished from vesicle fusion.

Therefore, single vesicle-vesicle assays have been developed to resolve individual fusion events and thus, get access to fusion intermediates, their formation and the kinetics. To setup these assays, one vesicle containing either t-SNAREs or v-SNAREs is immobilised via polyethylene glycol(PEG)/PEG-biotin-neutravidin on a glass substrate and the other vesicle (t-SNAREs or v-SNAREs) is added from solution and fusion is detected by total internal reflection fluorescence (TIRF) microscopy. Several studies showed lipid mixing by labelling each vesicle population with a donor and acceptor fluorophore and detecting the FRET efficiency (Diao et al. 2010, 2012; Kyoung et al. 2011; Yoon et al. 2006). Alternatively, only one vesicle population was labelled with a lipid analogue fluorescent dye in self-quenched concentration and an increase in fluorescence intensity was observed upon fusion (Kyoung et al. 2011). However, even though these lipid mixing assays provide information about the intermediate states of the fusion process and their kinetics, vesicle bursting as an underlying artefact of the signal could not be ruled out. Hence, content mixing was added to the assay by entrapping complementary labelled/unlabelled DNA strands into the vesicles (Diao et al. 2010, 2012; Kyoung et al. 2011) or a water-soluble fluorescent dye in one vesicle population (Kyoung et al. 2011). From the read out of the fluorescence signals, one could conclude that content mixing occurred. In these studies, the fusion kinetics turned out to be faster than those observed in bulk assays.

Both bulk fusion assays as well as single vesicle-vesicle assays are based on two highly curved vesicle populations fusing with each other. Such vesicle-vesicle assays can suffer from this non-physiological geometry. At the presynaptic site, a highly curved synaptic vesicle fuses with a rather planar presynaptic membrane. Hernandez et al. (Hernandez et al. 2014) have shown that if two vesicles fuse, the vesicle size i.e., the curvature of the membranes, greatly influences the number of SNAREs required for maximum lipid mixing. Considering the geometry at the presynaptic site, it would be much more desirable to establish a system with a planar bilayer geometry to which a single vesicle can fuse. Of course, this approach does not rule out the presence of proteins inducing local membrane curvature such as synaptotagmin (Hui et al. 2009; McMahon et al. 2010). To develop such a fusion assay, planar supported lipid bilayers (SLBs) with reconstituted t-SNAREs have been exploited, which were produced by direct vesicle adsorption and fusion to the planar substrate. Unlabelled SLBs containing the t-SNAREs, to which labelled proteoliposomes (v-SNARE) were added, were analysed by TIRF microscopy to observe fusion. However, the initial attempts of single-vesicle fusion to planar supported bilayers resulted in SNAP-25-independent fusion reactions (Bowen et al. 2004; Liu et al. 2005) and were Ca^2+^-dependent even though the system lacked synaptotagmin (Fix et al. 2004). With a content release assay, Wang et al. (2009) found that vesicles rather ruptured than transferred their content across the target membrane. These observations might be explained by the reduced protein mobility in SLBs (Brunger et al. 2015) as a result of short-range interactions between the proteins and the support (Kyoung and Sheets 2008). To increase the membrane-support distance, Karatekin et al. (2010) produced planar membranes via direct vesicle adsorption and fusion but included a polymer cushion between the membrane and the support. They labelled the SLBs containing syntaxin 1A and SNAP-25 to investigate the heterogeneity and fluidity of the membrane before fusion. To this membrane, synaptobrevin 2-containing vesicles were added by a microfluidic flow system.

Tamm and co-workers used a different approach to build up the supported bilayer. They produced SNARE-containing lipid bilayers by a two-step Langmuir–Blodgett/vesicle fusion procedure (Domanska et al. 2009) and reconstituted a preassembled SNARE complex (ΔN49 complex) (Pobbati et al. 2006) composed of syntaxin 1A, SNAP-25 and a fragment of synaptobrevin 2 (aa 49–96). Lipid mixing with synaptobrevin 2-doped vesicles occurred within tens of milliseconds. With this assay in hand, the group was able to analyse in great detail the docking and fusion efficiencies and their kinetics as a function of the lipid composition (Domanska et al. 2010; Kiessling et al. 2010; Kreutzberger et al. 2016). They extended their system also to a content release assay, in which the content from a vesicle with an entrapped soluble dye was released into the small cleft between the substrate and the SLB (Kiessling et al. 2010; Kreutzberger et al. 2015).

As there are a number of drawbacks associated with current state-of-the-art membrane architectures used to investigate fusion processes, there is still a demand for improved and alternative systems overcoming these disadvantages. It is desirable to establish a planar and continuous lipid bilayer with laterally mobile membrane components that is long-term stable and provides large second aqueous compartments underneath the target membrane that can take up the vesicle’s content. If these membranes are accessible to microscopy techniques, they would allow for the detection of single vesicle fusion events in a geometry that nicely resembles the situation occurring at the presynaptic membrane. One alternative membrane system to SLBs that might suffice these requirements are pore-spanning membranes (PSMs) (Mey et al. 2012; Reimhult and Kumar 2008; Warkiani et al. 2013; Zagnoni 2012). For a short summary of the common fusion assays found in literature along with seminal references, the reader is referred to Table 1.

**Table 1:** Summary of selected in vitro fusion assays discussed in the paragraph “in vitro fusion assays” using neuronal SNAREs

Model system	Literature
Bulk assay	Weber et al. (1998)
Adhered vesicle + vesicle	Yoon et al. (2006)
SLB + vesicle	Fix et al. (2004)
Langmuir-SLB + vesicle	Domanska et al. (2009)
PEG-SLB + vesicle	Karatekin et al. (2010)
PSM + vesicle	Schwenen et al. (2015)

### Pore-spanning membranes (PSMs)

**Fig. 2:**
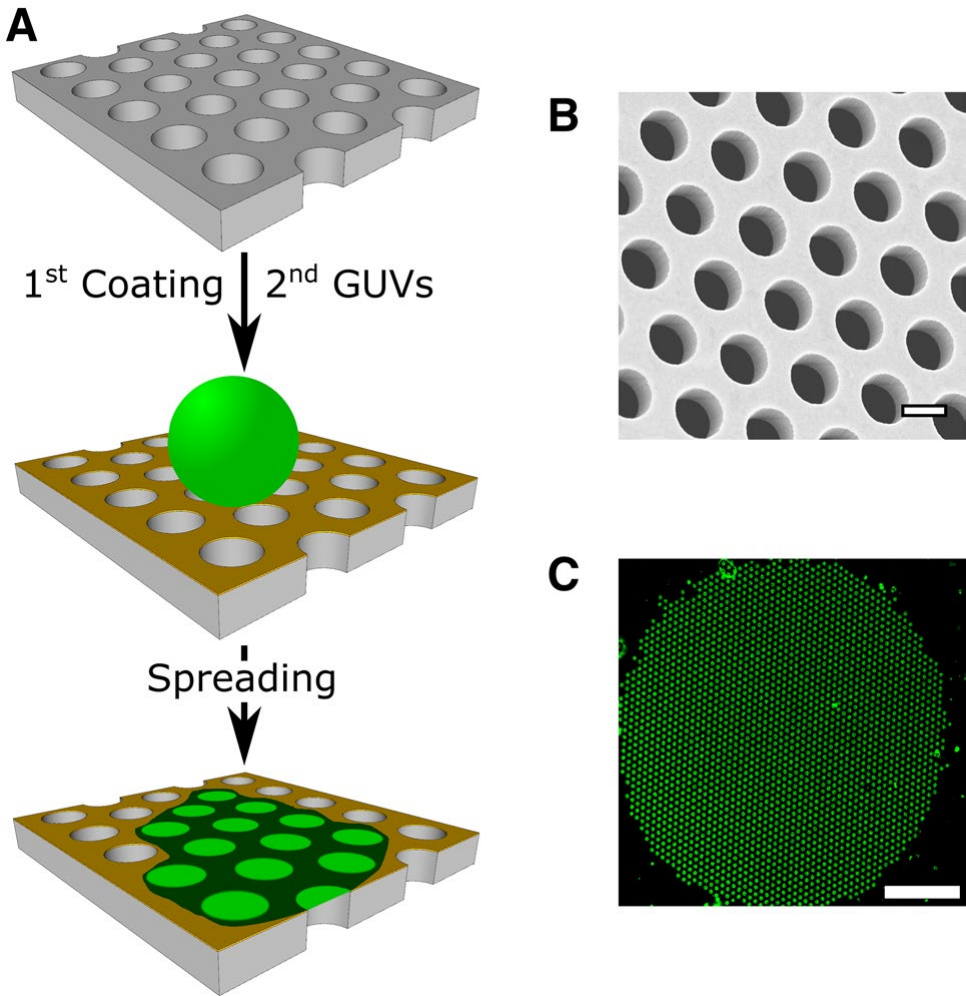
**a** Schematic drawing of the preparation procedure of a PSM on a gold/6-mercaptohexanol-functionalised porous silicon substrate. **b** Scanning electron micrograph of a porous silicon substrate with pore diameters of 1.2 μm. Scale bar: 1 μm. **c** Fluorescence micrograph of a PSM patch composed of DOPC/POPE/POPS/cholesterol (5:2:1:2) and doped with OregonGreen-DHPE. Scale bar: 20 μm. Adapted from (Schwenen et al. 2015)

PSMs have been shown to be mechanically robust and long-term stable (Römer et al. 2004), and the lipids in the PSMs are laterally mobile (Spindler et al. 2018; Weiskopf et al. 2007). As they are deposited on an open pore array, the membranes are accessible from both sides and provide enough space for the incoming lipid material during the fusion process (Höfer and Steinem 2011), while the aqueous space on either side of the membrane mimics more closely the in vivo conditions. To produce PSMs with the ability to reconstitute proteins, a method was developed based on spreading giant unilamellar vesicles (GUVs) on functionalised porous substrates (Fig. 2a). Si/Si_3_N_4_ or Si/SiO_2_ substrates with micrometre-sized pores arranged in a hexagonal array and a typical surface porosity of 35–40% are used. Figure 2b shows a scanning electron micrograph of a porous silicon substrate with pore diameters of 1.2 μm. The top part of the porous substrate is functionalised with a thin 30–40 nm gold layer, onto which 6-mercaptohexanol (6-MH) can be chemisorbed rendering the surface hydrophilic. This functionalisation allows individual GUVs to spread and form PSMs (Kaufeld et al. 2015; Kocun et al. 2011; Mey et al. 2012; Schütte et al. 2017). The resulting PSM patches with the size of the area of a single GUV (Fig. 2c) have a symmetric lipid composition and float on a thin aqueous layer ensuring high lateral mobility of the lipids. To define the lipid composition and to reconstitute proteins such as SNAREs into PSMs, GUVs with the corresponding membrane composition need to be produced, which can be a bottle-neck of the procedure. If fluorescently labelled lipid dyes are inserted into the GUVs, the resulting PSMs can be readily visualised by confocal fluorescence microscopy using an upright microscope.

A typical fluorescence micrograph of a PSM doped with a green fluorescent dye is shown in Fig. 2c. The fluorescence micrograph shows regions of high fluorescence intensity, which are membranes that span the pores (freestanding PSMs, f-PSMs), while the lipid fluorescence is quenched on the gold-coated supported parts (supported PSMs, s-PSMs) (Acuna et al. 2012; Chi et al. 2008).

### Single vesicle fusion experiments on PSMs

**Fig. 3:**
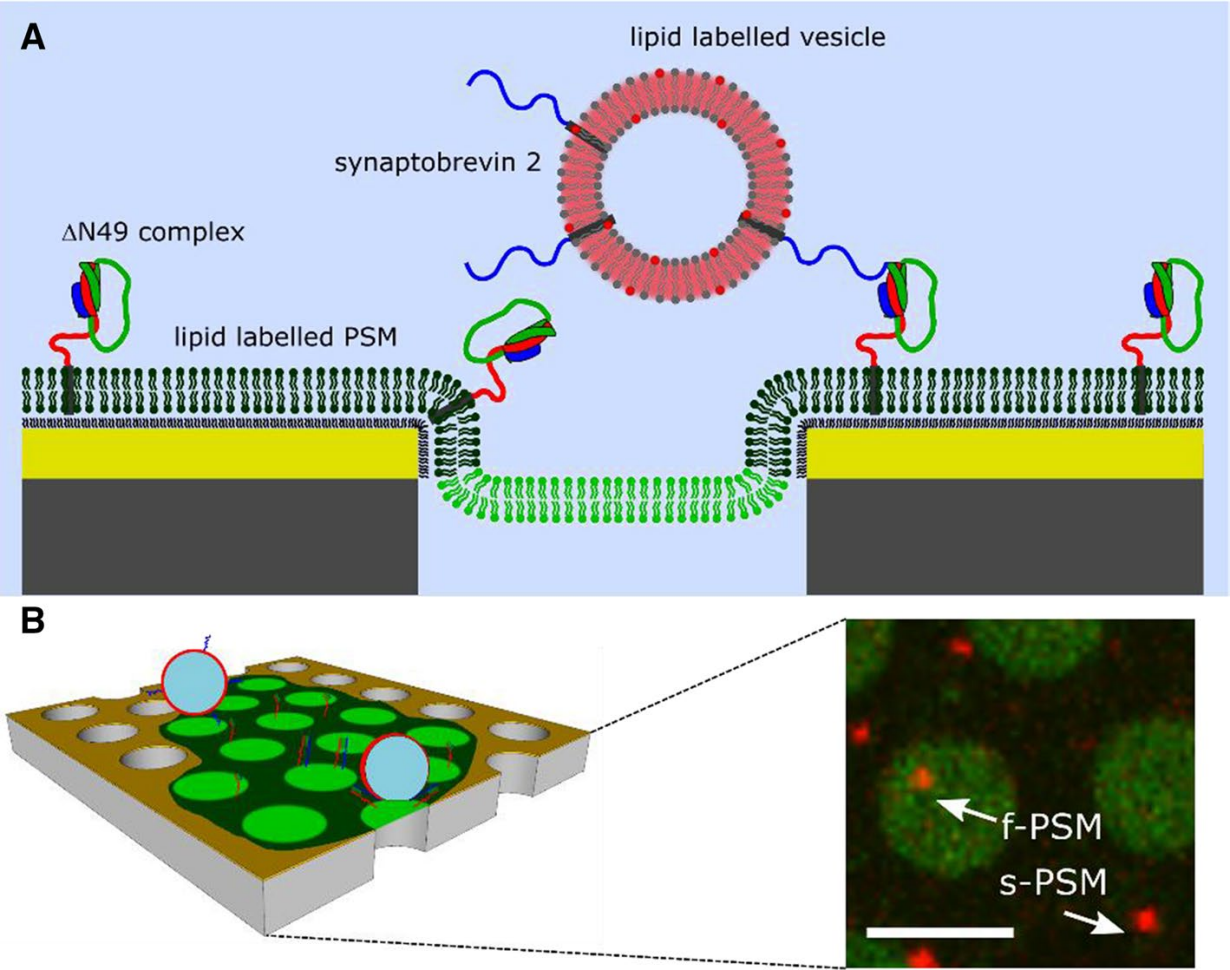
**a** Schematic drawing of the fusion setup (not drawn to scale). **b** Three-dimensional representation of the PSM with reconstituted t-SNAREs to which lipid-labelled vesicles containing the v-SNAREs are docked (left). The fluorescence micrograph (right) shows a PSM composed of DOPC/POPE/POPS/cholesterol (5:2:1:2) labelled with Atto488-DPPE and doped with the ΔN49 complex to which vesicles with the same lipid composition but doped with TexasRed-DHPE and synaptobrevin 2 were docked, either to the f-PSM or the s-PSM. Scale bar: 5 µm. Adapted from (Kuhlmann et al. 2017)

For a fusion experiment, the minimal machinery of neuronal fusion is reconstituted into the membranes. The SNAREs (t-SNAREs) syntaxin 1A and SNAP-25a (“a” indicates that all cysteine residues are mutated to serine residues) are reconstituted into the PSMs, while synaptobrevin 2 (v-SNARE) is reconstituted into the vesicles. This arrangement resembles the geometric membrane situation at the presynaptic site. In our studies, we use the preassembled syntaxin 1A/SNAP-25a/synaptobrevin 2 (residues 49–96) complex (1:1:1) termed the ΔN49 complex (Pobbati et al. 2006) (Fig. 3) as also used in the studies on SLBs by the Tamm group (Domanska et al. 2009). To analyse whether the SNAREs are reconstituted into PSMs and are laterally mobile, first fluorescently labelled syntaxin derivatives were used, namely Atto647N (Atto647N-syntaxin 1-transmembrane domain (TMD) and syntaxin 1A labelled with Alexa488 via a cysteine residue (Alexa488-syntaxin 1A). Both the fluorescence of the Atto647N-syntaxin 1-TMD and that of Alexa488-syntaxin 1A were observed in the f-PSMs indicating successful reconstitution of the transmembrane peptides in the bilayers (Schwenen et al. 2015). Whereas the fluorescence intensity of Atto647N-syntaxin 1-TMD was fully homogeneous throughout the f-PSMs, the fluorescence of Alexa488-syntaxin 1A was slightly inhomogeneous.

In literature, it is described that syntaxin 1A tends to cluster, driven by homotypic protein–protein interactions (Milovanovic et al. 2015; Milovanovic and Jahn 2015; Sieber et al. 2006, 2007). Prerequisite for the formation of a fusion complex during fusion of a vesicle with the planar bilayer is the lateral mobility of the proteins in the plane of the PSMs. By means of fluorescence correlation spectroscopy, we determined the diffusion constants of the transmembrane peptides. The results clearly demonstrated that the peptides are mobile in the f-PSMs (Schwenen et al. 2015). While for Atto647N-syntaxin 1-TMD, a diffusion constant in the f-PSMs of 3.4 ± 0.2 µm^2^/s was determined, Alexa488-syntaxin 1A exhibited a diffusion constant of 2.3 ± 0.2 µm^2^/s in the f-PSMs in good agreement with diffusion constants found for syntaxin 1A in GUVs (Bacia et al. 2004).

**Fig. 4:**
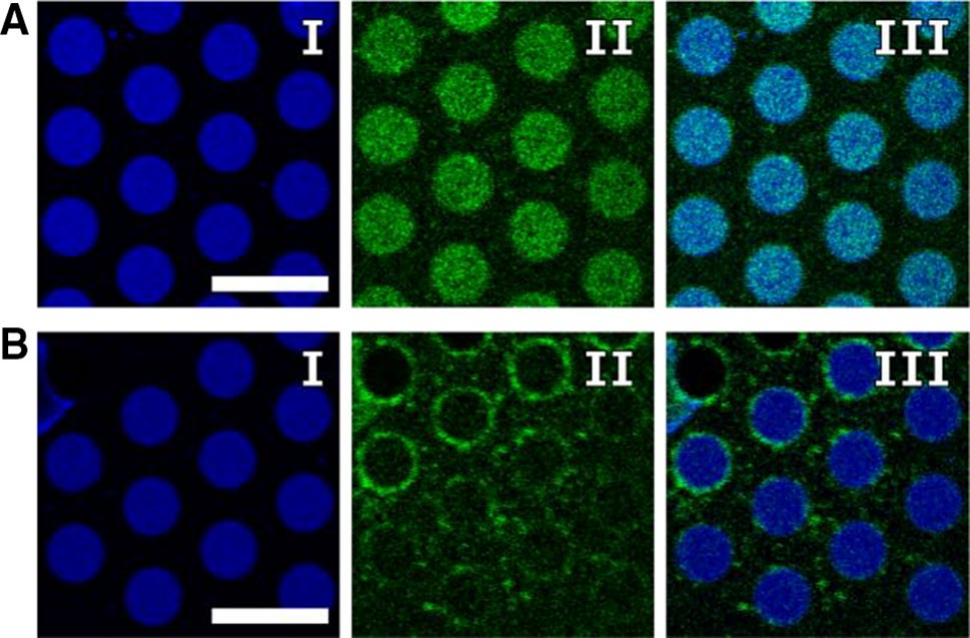
Fluorescence micrographs of Atto390-DPPE labelled PSMs (**a–b**, I) and reconstituted ΔN49-Atto488 complex (**a**–**b**, II) including the overlays of both channels (III). **a** Homogeneous fluorescence intensity of the protein is visible in the f-PSM. Scale bar: 10 μm. **b** The majority of protein fluorescence intensity is observed at the pore edges with some fluorescence spots in the s-PSM. Scale bar: 10 μm

As we observed a fluorescence inhomogeneity in case of syntaxin 1A, we asked the question, whether the reconstituted ΔN49 complex is also inhomogeneously distributed within the PSMs. To illuminate this aspect in more detail, we labelled the ΔN49 complex using a synaptobrevin 2 fragment (aa 49–96) that harboured a S79C mutation (ΔN49-Atto488). The single cysteine residue was labelled with Atto488 maleimide and then assembled with syntaxin 1A and SNAP-25a. After reconstitution into GUVs and spreading onto the porous substrates, fluorescence micrographs were taken (Fig. 4a/b). While the lipids were homogeneously distributed within the f-PSMs (Fig. 4a/b, I), the ΔN49 complex was found to be either homogeneously distributed (Fig. 4a, II) or it was more localised at the pore edges (Fig. 4b, II).

One explanation for this finding could be the topography of the PSMs. PSMs are not completely flat but bend at the pore edges as has been visualized by scanning ion conductance micrographs (Böcker et al. 2009; Schütte et al. 2017). This bend membrane structure results from the interaction between the lipids of the bilayer and the 6-mercaptohexanol self-assembled monolayer (6-MH SAM) on the gold surface (Fig. 3a). In the centre of the pore, the PSMs are flat presumably as they experience a certain pre-stress in the mN/m-range (Janshoff and Steinem 2015; Kocun et al. 2011; Kuhlmann et al. 2014). It is well conceivable that this membrane geometry, as well as the broad distribution of the ΔN49 complex concentration in the individual GUVs (Mühlenbrock et al. 2020) influence the observed differences in the protein distribution.

Once the PSMs with reconstituted ΔN49 complex are established, synaptobrevin 2-doped vesicles labelled with a red fluorescent dye can be added to observe single vesicle fusion events. As the porous silicon substrates are non-transparent, an upright confocal fluorescence microscope is required with a water immersion objective. An upright microscopy setup is essential to be able to observe vesicles that dock to the pore rims, which would be undetectable with an inverted microscope. Confocality is needed in this case to fade out the planes containing vesicles in solution. Using an overlay of the red and green fluorescence channel, as exemplarily shown in Fig. 3b, the specific docking of vesicles to the f-PSMs and s-PSMs can be observed in the fluorescence micrograph. The specificity of docking was proven by a control experiment. The SNARE-binding site of the ΔN49 complex was incubated with the soluble synaptobrevin 2 fragment (aa 1–96) that is known to block fusion before protein reconstitution. No docking of synaptobrevin 2-doped vesicles on these PSM was observed (Hubrich et al. 2019; Mühlenbrock et al. 2020).

We distinguished between vesicles docked to the f-PSMs and those that docked to the s-PSMs. In our first study, we simply used geometric considerations extracted from the fluorescence micrographs of the PSMs i.e., we distinguished the highly fluorescent f-PSMs from the dark s-PSMs and used a grid to define the two different areas. This approach resulted in about 50% of vesicles docked to either part of the PSM with very similar fusion kinetics and fusion intermediates (Schwenen et al. 2015). However, in a follow-up study (Kuhlmann et al. 2017), we found that vesicles docked to the f-PSMs were mobile, while vesicles docked to the s-PSMs were instantaneously immobile. Vesicles that docked to the edges of the f-PSMs and are thus partially in contact to the pore rim are also immobile. An assignment of the position of a docked vesicle based on this mobility criterion led us conclude that vesicles highly favour to dock to the s-PSMs (99%) either by directly docking to the pore-rim area or docking to the f-PSM, however with a subsequent immobilisation at the s-PSM, preferably at the edges between f-and s-PSMs. We suggest that the preferential localisation of the ΔN49 complex at the pore edges (Fig. 4b, II) is at least in part responsible for this finding. The fact that vesicles first dock to the f-PSMs and then immobilise at the edges of the s-PSM was independently observed by Ramakrishnan et al. (2019). They also observed t-SNARE aggregates at the pore edges and concluded that the immobility of the vesicles is a result of immobile t-SNAREs. However, as they used an inverted microscope setup in conjunction with the opaqueness of the silicon substrate, they could not observe vesicles docked on top of the s-PSMs and hence were not able to provide quantitative data about the ratio of vesicles docked to the f-PSMs and s-PSMs.

Besides the preferential location of the proteins at the pore edges, also the overall mobility of the membrane components adds to the observed immobility of the docked vesicles on the s-PSMs. The diffusion coefficients of the lipids and peptides are by a factor of 2–4 lower on the s-PSMs compared to the f-PSMs (Kuhlmann et al. 2017; Mühlenbrock et al. 2020; Schwenen et al. 2015). Moreover, we hypothesise that a conformal contact of the vesicle with the membrane on the gold-covered support with a large Hamacker constant, further immobilises the vesicles (Kuhlmann et al. 2017).

### Single vesicle lipid mixing on s-PSMs

**Fig. 5:**
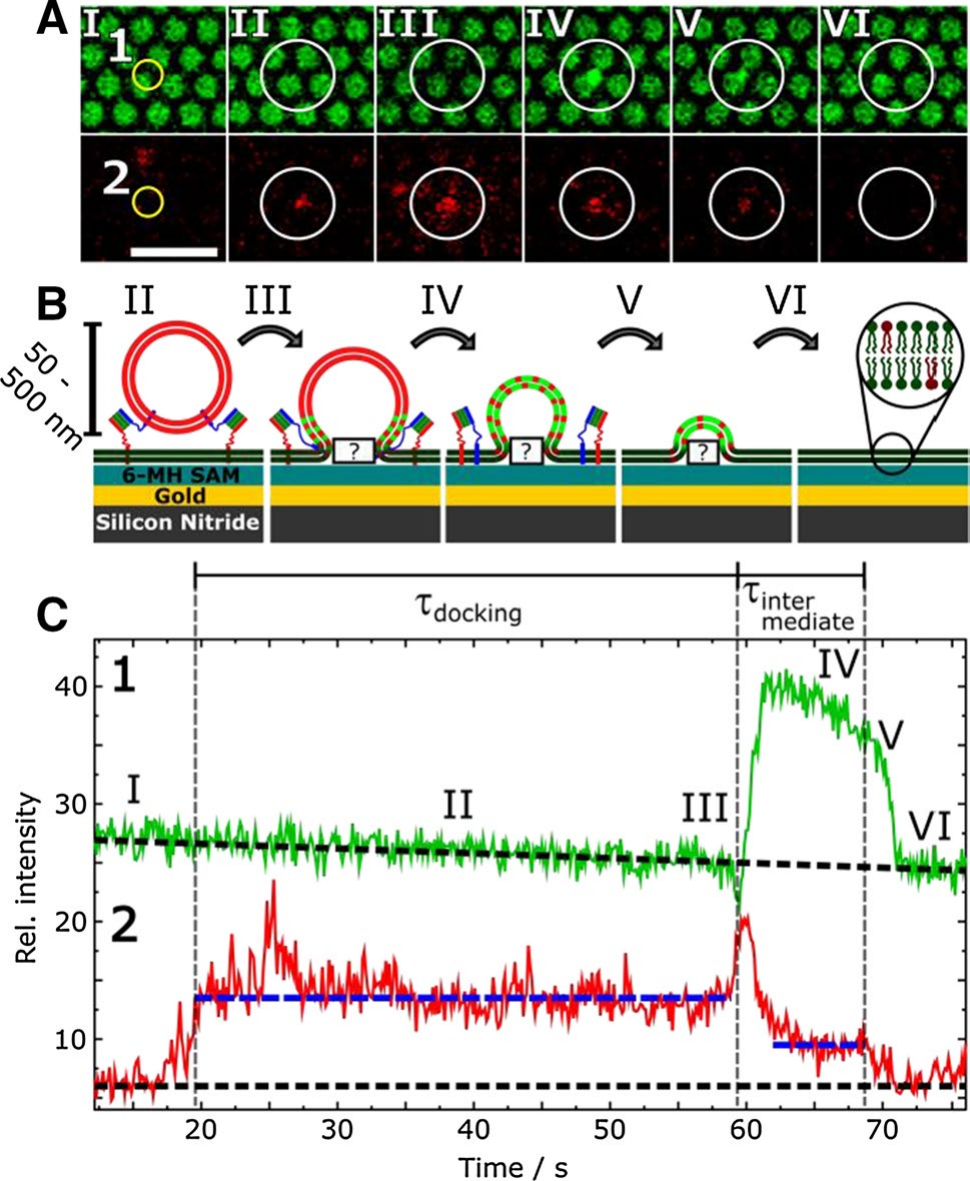
**a** Time lapse fluorescence images of a single vesicle fusion event of a large unilamellar vesicle doped with full length synaptobrevin 2 (lower panel, 2) with a PSM containing the ∆N49 complex (upper panel, 1). The region of interest (ROI) used to read out fluorescence intensities is highlighted with a yellow circle while the white circle serves as a guide to the eye to identify the region in which the vesicle docks and fuses; scale bar: 5 µm. **b** Schematic cross section of the possible fusion pathway of the vesicle fusing with the s-PSM and (**c**) corresponding fluorescence intensity time trace of the PSM (1, green) and vesicular membrane (2, red). Dashed black lines highlight the baselines while dashed blue lines highlight the different levels of vesicle fluorescence intensity. Upon docking to the PSM the red fluorescence of the vesicle is detected in the ROI (I/II, red). Upon lipid mixing of the outer leaflets the red fluorescence (III, red) is increased due to a FRET between OregonGreen-DHPE and TexasRed-DHPE followed by a fast diffusion into the PSM (IV, red). Simultaneously, lipid molecules of the PSM diffuse into the 3D structure of the vesicle and are de-quenched (IV, green). Full fusion of the vesicle with the membrane results in a second decrease of vesicle fluorescence intensity to baseline level (V/VI, red). Concomitantly, the 3D structure of the vesicle collapses into the target membrane (V/VI, green). The time between docking of the vesicle and fusion of the presumably outer leaflets is defined as *τ*_docking_. The time span between outer and inner leaflet mixing is defined as *τ*_intermediate_. Adapted from (Schwenen et al. 2015)

While the docking process can simply be observed by reading out the red fluorescence intensity time traces of the vesicle by defining a region of interest (ROI) around the vesicle, the fusion process itself can be investigated in much more detail if both the red fluorescence as well as the green fluorescence of the PSM is monitored simultaneously. Time traces of the red and green channel recorded during the docking and fusion of a single vesicle with the PSM are shown in Fig. 5.

Upon docking of a vesicle, fluorescence intensity increases in the red channel (I/II) and remains at a constant level until lipid mixing of (presumably) only the two outer leaflets (III) leads to an increase in the PSM fluorescence intensity (green channel, III) due to lipid dye diffusion into the 3D structure of the docked vesicle and thus out of the quenching regime of the underlying gold surface (Chen and Knutson 1988; Chi et al. 2008) and a partial decrease in vesicle fluorescence (red channel) (III/IV). When using a FRET-pair as lipid markers (here either OregonGreen-DHPE or Atto488-DPPE and TexasRed-DHPE), we observed in some cases a short drop in PSM fluorescence intensity prior to dequenching as well as a short spike in vesicle fluorescence intensity before lipids diffuse into the PSM and fluorescence intensity drops (Fig. 5, red channel, IV). We suggest that these intermediate fluorescence intensity levels indicate a hemifusion state. In case of hemifusion that proceeds to full fusion, merging of the inner leaflets leads to full lipid mixing and drop of intensity to baseline level (Fig. 5, red channel, VI). In this particular case, collapse of the vesicle into the target membrane begins earlier than inner leaflet mixing (Fig. 5, red and green channel, V/VI).

In our first study (Schwenen et al. 2015), we defined criteria to distinguish between docking, intermediate states and full fusion, which were applicable in the following studies. However, in the first study, we overestimated the number of detaching vesicles after docking. If the vesicle docked resulting in an increase in red fluorescence intensity followed by a drop back to baseline level in one step without change in the green PSM fluorescence, this process was originally interpreted as detachment of the docked vesicle. However, it turned out in a follow up-study, where we used a higher time resolution and an adapted fluorescence intensity read out strategy (Kuhlmann et al. 2017) that these vesicles quickly fuse and do not detach. Indeed, vesicle detachment turned out to be an extremely rare event.

**Fig. 6:**
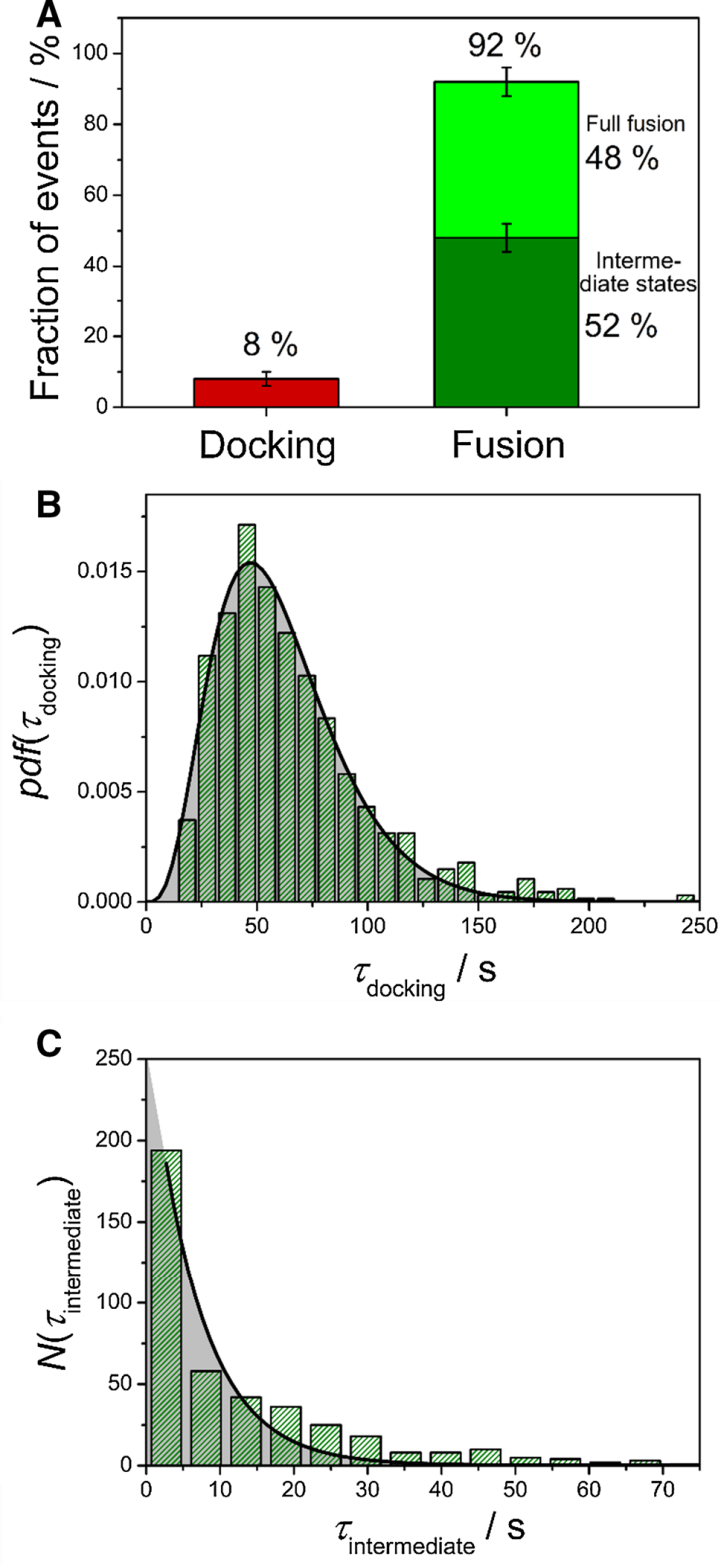
Statistical analysis of the fusion process of LUVs composed of DOPC/POPE/POPS/cholesterol/TexasRed-DHPE (50:19:10:20:1) and doped with synaptobrevin 2 (p/l 1:500) with PSMs composed of DOPC/POPE/POPS/PIP_2_/cholesterol/Atto488-DPPE (48:19:10:2:20:1) and doped with the ΔN49 complex (p/l 1:500). **a** Fusion efficiency. **b** Probability density function (pdf) of lifetimes of the docking state *τ*_docking_ with the result of fitting Eq. 1 to the data (black line) with *k*_1_ = 0.074 ± 0.003 s^−1^ and *N* = 4.5 ± 0.2 resulting in an average docking lifetime of *τ*_docking_ = 61 ± 5 s. **c** Histogram of the lifetimes of the intermediate states *τ*_intermediate_. Fitting Eq. 2 to the data (black line) results in a rate constant of *k*_2_ = 0.15 ± 0.02 s^−1^. Taken from (Hubrich et al. 2019)

With the criteria in hand, we were able to perform statistical analysis of the fusion efficiency as well as the kinetics. Figure 6 provides an example of the fusion statistics for PSMs doped with the ΔN49 complex, to which vesicles doped with synaptobrevin 2 were added. Under the given conditions, the fusion efficiency was, with 92% of all docked vesicles (Fig. 6a), quite high. A general model proposed by Floyd et al. (2008, 2010) was employed to shed some light on the docking lifetime that is defined as shown in Fig. 5c. In this model, the rate-limiting step from docking to the onset of fusion is not defined as a one-step transition but as a series of *N* hidden transitions with a single rate constant *k*_1_ for each transition (Eq. 1):

1 $$pdf\left( {\tau_{{{\text{docking}}}} } \right) = \frac{{k_{1}^{N} \cdot \tau_{{{\text{docking}}}}^{N - 1} }}{{{\Gamma }\left( N \right)}} \cdot {\text{exp}}\left( { - k_{1} \cdot \tau_{{{\text{docking}}}} } \right) ,$$

with Γ(*N*) being the gamma function. By fitting Eq. 1 to the dwell time distribution of docked vesicles (Fig. 6b), a rate constant of *k*_1_ = 0.074 ± 0.003 s^−1^ and *N* = 4.5 ± 0.2 resulting in an average docking lifetime of ${\stackrel{-}{\tau }}_{\mathrm{docking}}$= 61 ± 5 s was determined.

These docking lifetimes are astonishing large compared to docking lifetimes obtained in other fusion assays. Based on SLBs on glass substrates separated by PEG brushes, Karatekin et al. (2010) found docking lifetimes of 130 ms for the fusion of small unilamellar vesicles (SUVs) with reconstituted synaptobrevin 2 with the target membrane containing the syntaxin 1A/SNAP-25 complex. Their described reconstitution protocol might have included undesired syntaxin 1A/SNAP-25 (2:1) complexes (Pobbati et al. 2006). Kreutzberger et al. (2016) developed two protocols to control the formation of the desired syntaxin 1/SNAP-25 (1:1) complex and analysed the docking lifetimes in comparison to the reconstituted ΔN49 complex, which was also used in case of the PSMs. In their assay composed of SLBs with reconstituted t-SNAREs, to which vesicles doped with synaptobrevin 2 were added, they found docking lifetimes in the ten milliseconds range independent of the chosen t-SNARE complex (Domanska et al. 2009, 2010; Kreutzberger et al. 2016). The same ten milliseconds docking lifetime was found for a fusion assay based on GUVs doped with the ΔN49 complex, to which single large unilamellar vesicles (LUVs) doped with synaptobrevin 2 were fused (Witkowska and Jahn 2017). From these results one can conclude that the t-SNARE complex is not the decisive parameter for the differences in docking lifetimes. However, it remains still a bit puzzling why there is no difference in docking lifetimes observed for the ΔN49 complex compared to the syntaxin 1A/SNAP-25 (1:1) complex, as bulk anisotropic measurements identified the replacement of the synaptobrevin 2 fragment from the ΔN49 complex as the rate limiting step between docking and fusion with a half lifetime of about 20 s (Pobbati et al. 2006). This displacement of the synaptobrevin 2 fragment would be in the time window of the docking lifetimes observed on PSMs but apparently does not become visible in the fusion processes on supported membranes. Indeed, the question still remains whether all assays measure SNARE specific docking and SNARE-induced fusion and which other factors, such as the different surfaces (gold vs. glass) contribute to the docking lifetimes.

To evaluate the stability of the intermediate states, we generated histograms for their lifetimes (Fig. 6c). τ_intermediate_ is defined as depicted in Fig. 5c. Equation 2 was fit to the histogram to determine *k*_2_, the rate constant for the onset of the collapse of the three-dimensional structure into the PSM:

2 $$N\left( {\tau_{{{\text{intermediate}}}} } \right) = N_{0} \cdot {\text{exp}}\left( { - k_{2} \cdot \tau_{{{\text{intermediate}}}} } \right),$$

with *N*_0_ being the total number of instable intermediate structures. *k*_2_ = 0.15 ± 0.02 s^−1^ means that the average lifetime of the three-dimensional structure before the onset of full fusion is *k*_2_^−1^ = 6.7 ± 0.9 s.

The described lipid mixing assay nicely demonstrates that, based on a statistical analysis of individual vesicle fusion events, quantitative information can be gathered about the fusion process. However, one important information that this setup as well as other lipid mixing based fusion assays lacks is, when a fusion pore opens and to what extent the vesicular content is released upon fusion. Hence, we envisioned to expand our PSM system to a content release assay, exploiting the second aqueous compartments underneath the PSMs.

### Single vesicle content release on s-PSMs

**Fig. 7:**
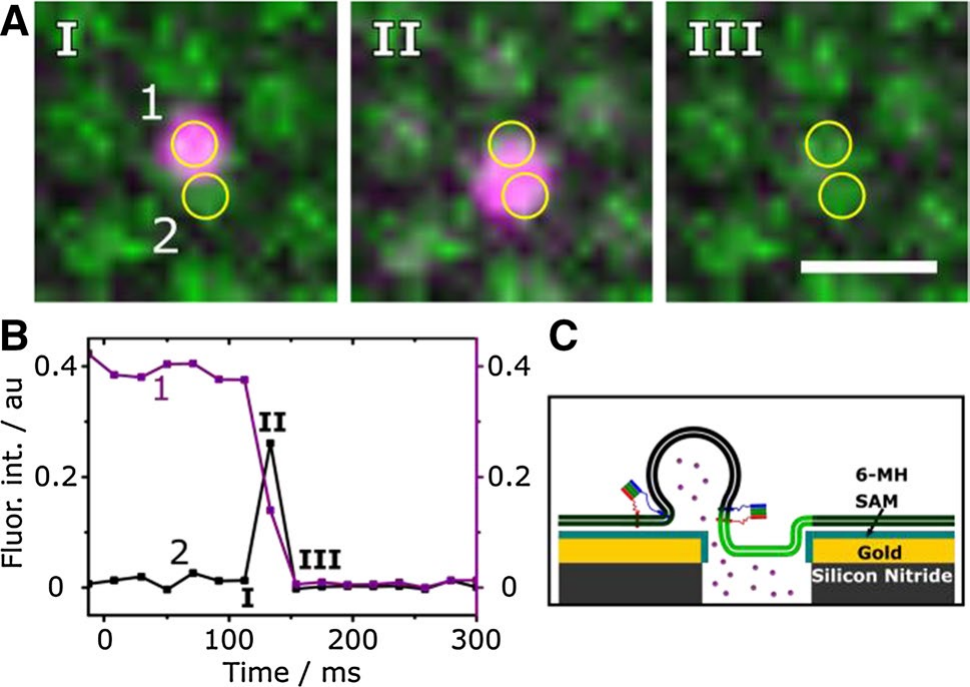
**a** Time lapse fluorescence micrographs of a fusing vesicle (magenta) that transfers its content across the PSM. Yellow circles highlight the ROI of the vesicle (1) and the neighbouring aqueous compartment underneath the f-PSM (2); scale bar: 2 μm. **b** Fluorescence intensity time traces obtained from ROI 1 + 2. The docked vesicle (I) fuses and transfers its content (II) across the PSM and into the second aqueous compartment, thus leading to an increase in fluorescence intensity visible in ROI 2. **c** Schematic illustration of the process shown in *A* and *B*. Adapted from (Mühlenbrock et al. 2020)

To establish the system, vesicles doped with synaptobrevin 2 were filled with the water-soluble dye sulforhodamine B (SRB) at self-quenching concentrations. If the vesicle fuses with the PSM concomitant with fusion pore formation, SRB is released in the underlying aqueous compartments, and the fluorescence intensity is increased due to dye dilution. A time series of a fusing vesicle is depicted in Fig. 7. The vesicle, visible in the SRB fluorescence image (Fig. 7a), docks at the edge of the pore (Fig. 7c), as observed before, and releases its content in the adjacent aqueous compartment (Fig. 7a/b, ROI 2). If one reads out the fluorescence intensity directly at the site of vesicle docking, (Fig. 7a/b, ROI 1) the fluorescence intensity drops to baseline level.

**Fig. 8:**
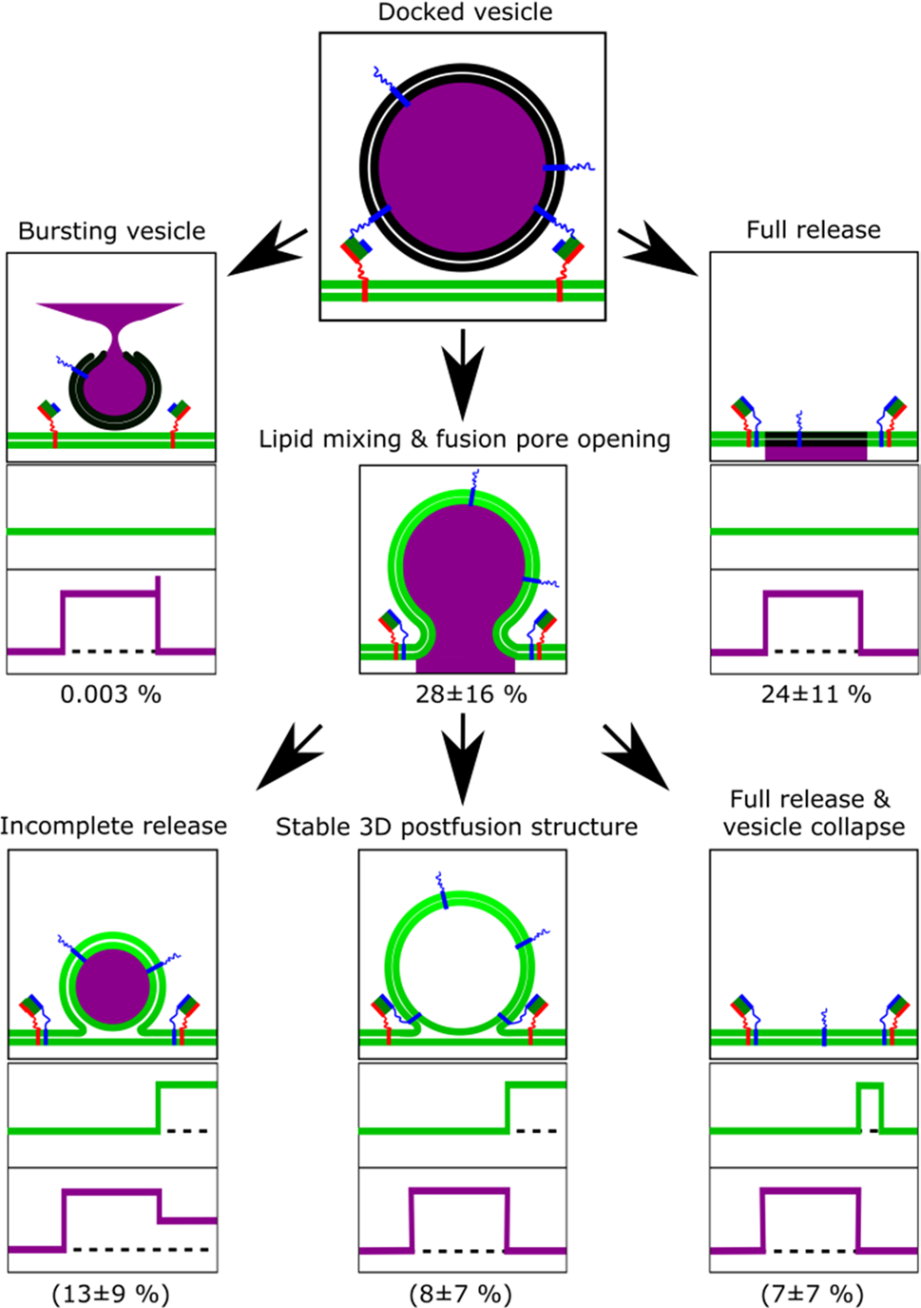
Different fusion pathways extracted from single vesicle fusion events with idealised fluorescence intensity time traces. Taken from (Mühlenbrock et al. 2020)

With dual colour fluorescence read out, the content release (SRB fluorescence) and the diffusion of lipids (Atto655-DPPE) from the PSM into the vesicular structure can be extracted for each individual fusion event. With these two fluorescence time traces (idealised fluorescence intensity time traces are shown in Fig. 8), it became possible to identify and quantify the different fusion intermediates and fusion pathways (Fig. 8).

Most of the docked vesicles released their content completely (75% of fusing vesicles). 24% of docked vesicles released their content without visible lipid diffusion via a fusion stalk, whereas 28% of docked vesicles underwent fusion with visible lipid mixing into an either stable or unstable 3D structure and/or showing an incomplete content release. A process where hemifusion occurred without fusion pore formation was negligible. If only lipid mixing was used to measure the fusion process, we assigned the observed intermediate state to a hemifusion state, if the fluorescence intensity of the vesicle membrane drops and PSM fluorescence increases to a constant level above the baseline. However, if a hemifusion diaphragm were really formed without a fusion pore, no content release would be observed. Taken the information from the content release assay into consideration, it appears to be more appropriate that the intermediate state is defined by a quick opening and closing of the fusion pore rather than a stable hemifusion state.

This result demonstrates that it is highly desirable to establish a fusion assay in which lipid mixing and content release can be observed simultaneously. For example Tamm and co-workers (Kiessling et al. 2013; Kreutzberger et al. 2017b) as well as others (Stratton et al. 2016) have extended their fusion assays based on SLBs to measure fusion pore formation. As there is no large aqueous space underneath the membrane, they rely on a characteristic diffusion behaviour of the released vesicular content to define whether the vesicle formed a fusion pore or burst. It is assumed that only if a fusion pore is formed resulting in the dye to be released inside the narrow cleft between membrane and support, a two-dimensional diffusion behaviour of the dye can be observed. Thus, if a two-dimensional diffusion model fits the data, it is concluded that a fusion pore has been formed.

By reading out the SRB fluorescence (fusion pore formation) and Atto655-DPPE fluorescence (lipid mixing) we were furthermore able to quantify the time difference between lipid diffusion into the 3D structure of the vesicle and content release. With the given time resolution of about 20 ms, the two processes appear to be quasi-simultaneously. This finding is in agreement with previous reports (Ramakrishnan et al. 2018; Stratton et al. 2016).

### Single vesicle fusion with f-PSMs

**Fig. 9:**
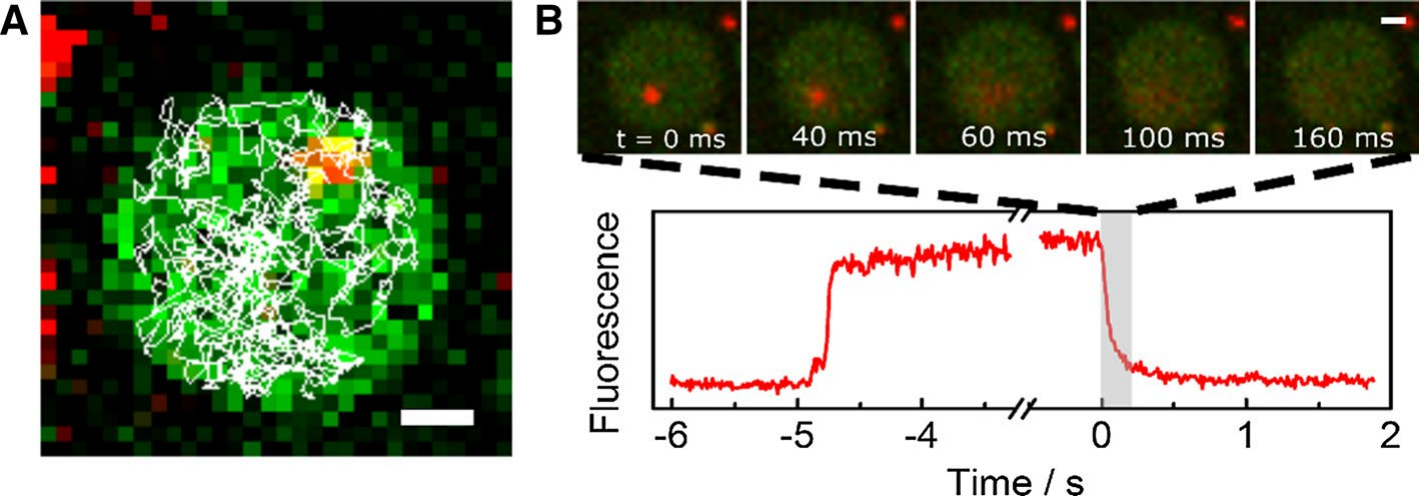
**a** Fluorescence micrograph of a f-PSM (green) together with the trajectory (white) of a mobile docked vesicle fusing with the f-PSM. **b** Fluorescence micrographs of the fusion process and respective fluorescence intensity time trace of the fusion event; scale bars: 1 µm. Adapted from (Kuhlmann et al. 2017)

The majority of fusion events turned out to occur at the s-PSMs concomitant with the immobility of the docked vesicle. However, we also observed vesicles that docked to the f-PSM and remained fully mobile within the f-PSM (Fig. 9a) till they proceeded to fusion (Fig. 9b). To evaluate their diffusion behaviour, trajectories of the diffusing vesicles were read out. From the mean square displacements, a mean diffusion coefficient of 0.42 ± 0.15 μm^2^/s was calculated. Compared to the diffusion constant of syntaxin 1A in the f-PSM, the diffusion coefficient of the docked vesicles is by a factor of five smaller. A close contact between vesicle and membrane and the displacement of the intermembrane water layer as well as multiple interacting SNARE-complexes might be responsible for the reduced diffusion coefficient compared to that of a single SNARE.

Ramakrishnan et al. (2018) saw similar results for vesicles docking and fusing with the f-PSM in their lipid mixing based assay. While not quantified, they report on a slowing down in diffusion of the docked vesicle until it seems to be almost immobile and explained this finding by an increase in the number of SNARE-complexes formed during the docking process.

### Chromaffin granules and PSMs

**Fig. 10:**
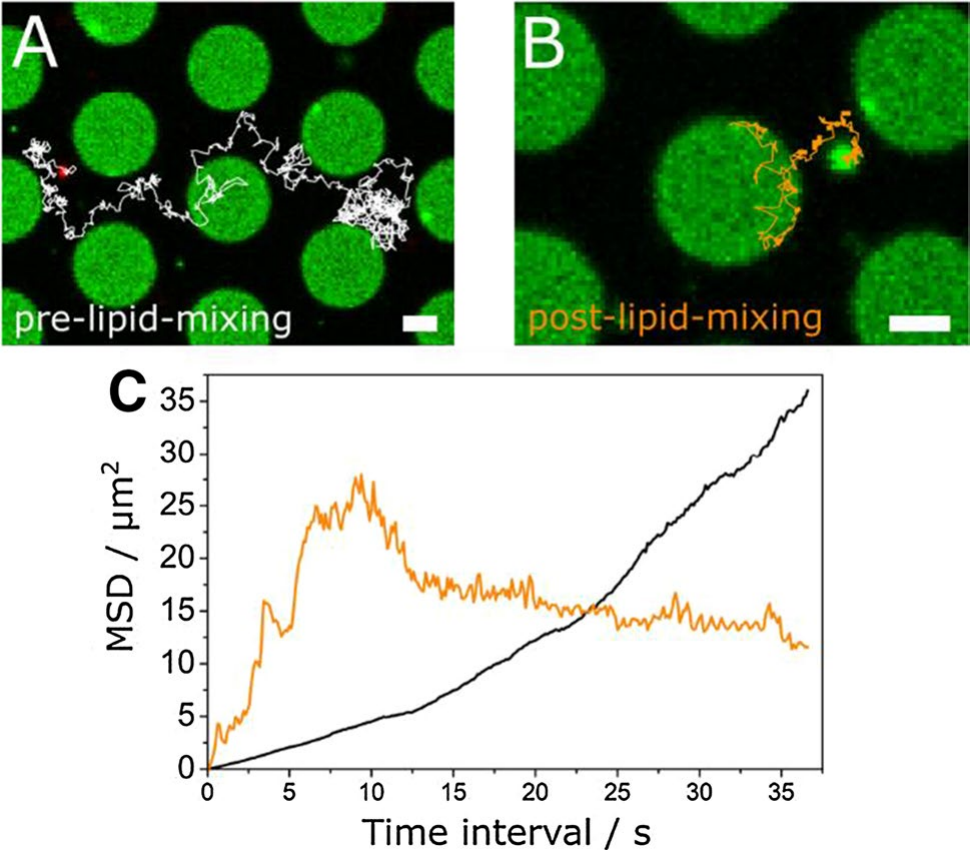
Fluorescence micrographs of a CG diffusing on the PSMs crossing pore boundaries (**a**) before and (**b**) after the onset of lipid mixing. Diffusion trajectories are depicted as white or orange lines, respectively. Scale bars: 2 µm. Mean square displacements (MSD) of the trajectories of CG diffusion in the pre-lipid mixing state (black line) and post-lipid mixing state (orange). Adapted from (Hubrich et al. 2019)

Synthetic vesicles containing a few types of lipids and synaptobrevin 2 are a very minimalistic system to investigate neuronal fusion. We thus asked the question whether synthetic vesicles doped with synaptobrevin 2 behave differently compared to natural vesicles. We replaced the synthetic vesicles with chromaffin granules (CGs), while keeping the PSMs doped with Atto488-DPPE and the reconstituted ΔN49 complex. CGs were isolated from the adrenal medulla of bovine glands using a continuous sucrose gradient for final purification (Park et al. 2012) and labelled with the lipophilic dye DiD-C_18_. CGs naturally containing the v-SNARE synaptobrevin 2 (Höhne-Zell et al. 1994) were added to the PSMs and their docking and fusion was investigated by dual colour confocal fluorescence microscopy (Hubrich et al. 2019). Once a CG has specifically docked on the PSM, it diffused onto the f-PSM as well as on the s-PSM and was even able to cross the borders (Fig. 10a). This finding was characteristic for CGs and was not observed if synthetic SNARE-doped vesicles were bound to PSMs as described above. The synthetic vesicles were either mobile and confined to the pore if they were docked to the f-PSM (see Fig. 9a) or they were immediately immobile, if they had docked to the s-PSM independent of the lipid composition (Hubrich et al. 2019; Kuhlmann et al. 2017). As long as the CG appears red in the fluorescence micrograph, no lipid mixing has occurred indicating that the CG is attached to the PSM via the formation of *trans*-SNARE complexes without the formation of a fusion stalk (pre-lipid mixing state, pre-lm). Once lipid mixing starts, the CG turns green, as the green fluorophore from the PSM diffuses into the CG, while the red fluorophore diffuses out. We call this state the post-lipid mixing state (post-lm) (Fig. 10b). Of note, in this post-lipid mixing state, the CG is still fully mobile on the f-PSMs as well as on the s-PSMs.

From trajectories obtained either solely on the s-PSM or f-PSM we computed the mean square displacement (MSD) (Fig. 10c). From short time intervals of several 100 ms, where CG diffusion was found to be unhindered, the diffusion constant *D* was calculated from the slope. Individual trajectories of diffusing CGs only dwelling either on the f-PSMs or the s-PSMs before or after the onset of fusion resulted in mean diffusion coefficients on the f-PSMs of ${\bar{D}}_{\mathrm{f-PSM}}$ (pre-lm) = 0.34 ± 0.06 µm^2^/s and ${\bar{D}}_{\mathrm{f-PSM}}$ (post-lm) = 0.40 ± 0.13 µm^2^/s. These diffusion constants are quite similar to those obtained for synthetic vesicles on f-PSMs, which suggests that CG diffusion is not altered by the fusion state i.e., whether the particle is in the pre-lipid-mixing state or post-lipid-mixing state on f-PSMs. On s-PSMs, mean diffusion coefficients for CGs were slightly smaller with ${\bar{D}}_{\mathrm{s-PSM}}$ (pre-lm) = 0.12 ± 0.05 µm^2^/s and ${\bar{D}}_{\mathrm{s-PSM}}$ (post-lm) = 0.04 ± 0.03 µm^2^/s.

These results suggest that the full immobility observed for s-PSM-docked synthetic synaptobrevin 2-doped vesicles cannot be explained by fully immobile ΔN49 complexes in the s-PSMs (Kuhlmann et al. 2017). Another aspect that might be considered is the frictional coupling of the vesicle (Yoshina-Ishii et al. 2006) on the s-PSMs. Frictional coupling is expected to be reduced for CGs compared to synthetic vesicles owing to the large protein content in the membrane serving as a spacer between vesicle and PSM so that synthetic vesicles become fully immobile, while CGs remain mobile on the s-PSMs. This mobility of docked CGs on s-PSMs allowed us to further analyse their diffusion behaviour. We compared CG trajectories on f-PSMs and s-PSMs. While CGs (pre-lipid- and post-lipid-mixing state) diffused freely on f-PSMs, they stayed confined for a certain time period on s-PSMs and at the edges of the pores, before they continued to diffuse. From a detailed quantitative analysis of the trajectories, where we defined three (arbitrarily set) mobility states as a function of the position of the CG on the membrane in the pre-lipid-mixing state and post-lipid-mixing state, we concluded that the fusion state (pre-lipid-mixing and post-lipid-mixing) does not significantly influence the diffusion behaviour of CGs. However, the CG mobility was impacted on the support dependent on whether they were in the pre-lipid mixing or post-lipid mixing state. We found that CGs in the post-lipid mixing state were primarily found in the least mobile state, while CGs in the pre-lipid mixing state were equally found in all three mobility states. Apparently, in the post-lipid mixing states, the CGs couple more strongly to the support. To our knowledge, PSMs provide for the first time a planar artificial model membrane, on which full mobility of CGs has been observed. In CG fusion experiments on SLBs, the particles became immediately immobile after docking (Kreutzberger et al. 2017a).

In contrast to the different diffusion behaviour, the fusion process itself was very similar to what we have found for synthetic vesicles. We determined the docking lifetime of CGs bound to the PSMs and found a rate constant of *k*_1_ = 0.040 ± 0.004 s^–1^ with a number of hidden transitions of *N* = 3.2 ± 0.3 according to the Floyd model (Floyd et al. 2008). From these parameters, we calculated the docking lifetime ${\stackrel{-}{\tau }}_{\mathrm{docking}}$ = *k*_1_^−1^ · *N* = 80 ± 16 s. Compared to the observed fusion of CGs with GUVs (Witkowska and Jahn 2017) this value is much larger, but very similar to what we have found for the docking lifetimes of synthetic vesicles with PSMs (Schwenen et al. 2015) independent of the lipid composition. Along the same line, we found that the lifetime of the semi-stable intermediate fusion states is very similar (*k*_2_^–1^ = 4.2 ± 0.9 s) to that of the synthetic vesicles. These results suggest a consistent one-step mechanism for the decay of the intermediate fusion state.

In conclusion, our results show that the high protein content of the CGs in the membrane greatly influences the dynamics of the docked vesicles to the PSM but does not alter the fusion kinetics significantly. While the in vitro membrane models using synthetic vesicles with reconstituted synaptobrevin 2 (Karatekin et al. 2010; Kiessling et al. 2010) including our own experiments (Kuhlmann et al. 2017) capture the basic features of the fusion process itself, they apparently do not reflect the natural behaviour of docked and partially fused vesicles.

## Conclusions

Pore-spanning membranes are a versatile tool to measure SNARE-mediated single vesicle fusion events on a quasi-planar membrane geometry mimicking the situation at the synaptic bouton. These membranes are readily accessible by confocal fluorescence microscopy allowing for a high spatial and time resolution. In contrast to SLBs, PSMs provide freestanding bilayer parts that allow taking up the incoming lipid material and a large aqueous space underneath the membrane that can harbour the content of the fusing vesicle. Using dual colour fluorescence techniques enables one to simultaneously measure lipid mixing as well as content release providing the required information to unravel different fusion intermediates and fusion pathways. While there are a number of merits associated with this membrane system, there are, however also some drawbacks and challenges that need to be overcome in the future to optimise the fusion assay based on PSMs. It would be desirable to better control the reconstitution of the fusion proteins in GUVs required to generate PSMs. Moreover, the inhomogeneous distribution of the proteins as well as the frictional coupling of the vesicles on the s-PSMs need to be illuminated to get a better handle on the system.
